# Congenital Antral Web Presenting as Gastric Outlet Obstruction in Adulthood: A Case Report

**DOI:** 10.7759/cureus.107239

**Published:** 2026-04-17

**Authors:** Mohamad Al Ayoubi, Nada Saleh, Tasneem M Freijeh, Zeinab Shaito, Garo Hovsepian, Antoine Abi Abboud

**Affiliations:** 1 Gastroenterology, Lebanese University Faculty of Medicine, Beirut, LBN; 2 Internal Medicine, Lebanese University Faculty of Medicine, Beirut, LBN; 3 Gastroenterology and Hepatology, Lebanese Hospital Geitaoui, University Medical Center, Beirut, LBN; 4 Gastroenterology and Hepatology, Lebanese University Faculty of Medicine, Beirut, LBN

**Keywords:** endoscopic balloon dilation, endoscopic web incision, gastric antral web, gastric outlet obstruction, rare gastric disorders

## Abstract

Gastric antral web (GAW) is a mucosal diaphragm causing gastric outlet obstruction. The etiology of this rare condition is heterogeneous, and clinical presentation ranges from asymptomatic disease to abdominal distention, early satiety, and weight loss. Diagnosis, while requiring a high index of suspicion, can be made by upper endoscopy, the gold standard, which reveals a tight antral web. Therapeutic options can be endoscopic, using multiple methods such as balloon dilatation and endoscopic web incision, or a surgical approach when endoscopic techniques fail. We present a case of gastric outlet obstruction due to an antral web, managed endoscopically, highlighting the importance of considering GAW in adults with unexplained upper gastrointestinal symptoms.

## Introduction

Gastric antral web (GAW), also known as an antral mucosal diaphragm, is a rare abnormality of the gastric antrum that can cause gastric outlet obstruction [1]. It has two main etiologies: congenital and acquired [2]. The congenital form typically presents during infancy and is attributed to defects in embryogenesis, while the acquired form is usually secondary to peptic ulcer disease [3].

GAW may be asymptomatic or symptomatic. When symptoms occur, they are often vague and variable, depending on the size of the web [1,4]. Symptomatic cases can be managed either endoscopically or surgically [4].

We report a case of a 20-year-old female who presented to our gastroenterology department with a one-month history of vague abdominal pain, vomiting, belching, and bloating. On upper endoscopy, a large amount of fluid and retained food was observed in the pylorus, antrum, and fundus. Examination revealed a tight antral web, making intubation challenging; however, the diaphragm was successfully dilated during the procedure.

## Case presentation

A 20-year-old previously healthy female, not taking any medications and with a BMI of 24, presented with a one-month history of intermittent abdominal pain associated with nausea and vomiting, along with alternating episodes of diarrhea and constipation. She was hemodynamically stable on presentation, and the physical examination was unremarkable. Laboratory investigations, including complete blood count, metabolic panel, and liver function tests, were all within normal limits (Table 1).

**Table 1:** Laboratory results at initial outpatient evaluation CRP: C-reactive protein; SGOT: serum glutamic-oxaloacetic transaminase; AST: aspartate aminotransferase; SGPT: serum glutamic-pyruvic transaminase; ALT: alanine aminotransferase; GGT: gamma-glutamyl transferase; ALP: alkaline phosphatase

Test	Result	Reference range
Complete blood count		
White blood cell count	3.98×10^9^/L	(4.0-11.0)10^9^/L
Hemoglobin	14.6 g/dL	13.5-17.5 g/dL
Hematocrit	43.8%	41%-53%
Platelet count	239×10^9^/L	(150-400)×10^9^/L
Neutrophils	68%	40%-70%
Lymphocytes	23%	20%-45%
Biochemistry panel		
Creatinine	0.8 mg/dL	0.7-1.3 mg/dL
Sodium	143 mmol/L	135-145 mmol/L
Potassium	4.3 mmol/L	3.5-5.1 mmol/L
Chloride	103 mmol/L	98-107 mmol/L
Bicarbonate	28.1 mmol/L	22-29 mmol/L
SGOT (AST)	38 U/L	10-40 U/L
SGPT (ALT)	50 U/L	7-56 U/L
GGT	40 U/L	9-48 U/L
ALP	98 U/L	44-147 U/L
Bilirubin (total)	0.5 mg/dL	0.1-1.2 mg/dL
Bilirubin (direct)	0.2 mg/dL	0.0-0.3 mg/dL
Lipase	18 U/L	13-60 U/L
Amylase	56 U/L	30-110 U/L
CRP	3 mg/L	0-5 mg/L

A contrast-enhanced CT scan of the abdomen and pelvis showed no evidence of bowel obstruction, inflammatory pathology, or other acute intra-abdominal abnormalities.

**Figure 1:**
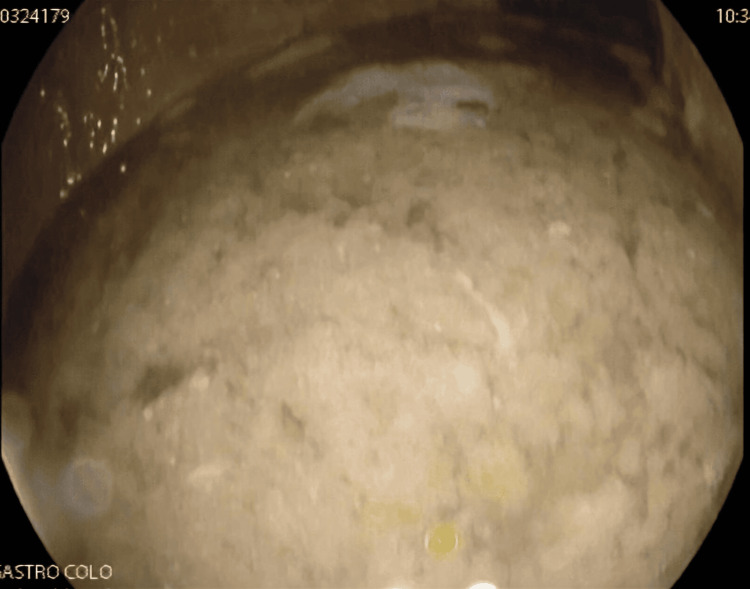
Upper gastrointestinal endoscopy showing a large amount of retained fluid and food debris within the gastric corpus

The patient underwent a comprehensive endoscopic workup. Ileocolonoscopy was performed to rule out inflammatory bowel disease (IBD) and was normal. Gastroscopy revealed a large amount of retained fluid and food debris in the gastric corpus and antrum (Figure 1).

**Figure 2:**
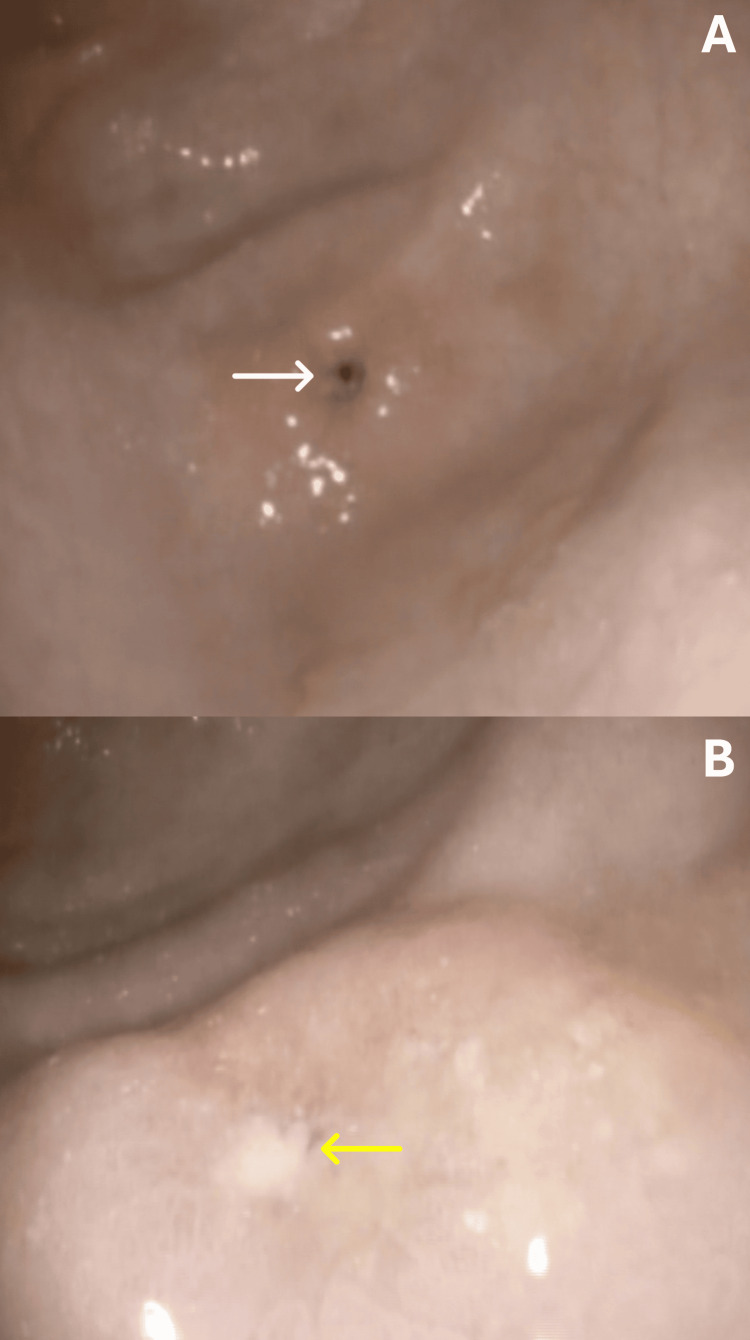
Endoscopic views showing a severely narrowed antral web (A, white arrow) and a pinpoint GAW (B, yellow arrow), causing gastric outlet obstruction GAW: gastric antral web

A severely narrowed, pinpoint antral web located approximately 3 cm above the pyloric ring was demonstrated, causing gastric outlet obstruction (Figures 2A, 2B).

**Figure 3:**
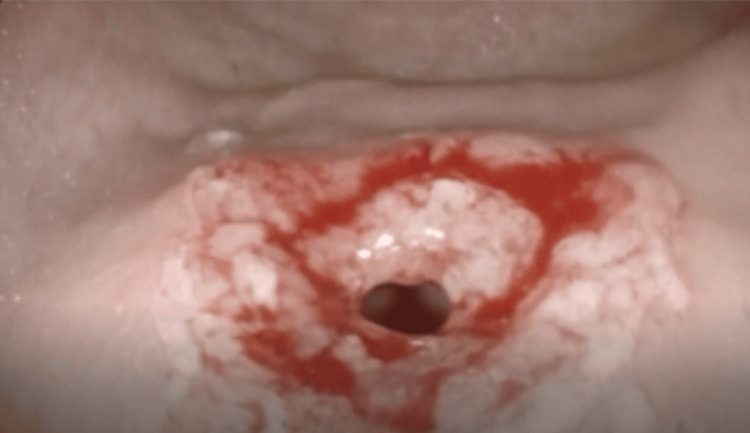
EBD was successfully performed, resulting in adequate widening of the antral web EBD: endoscopic balloon dilation

**Figure 4:**
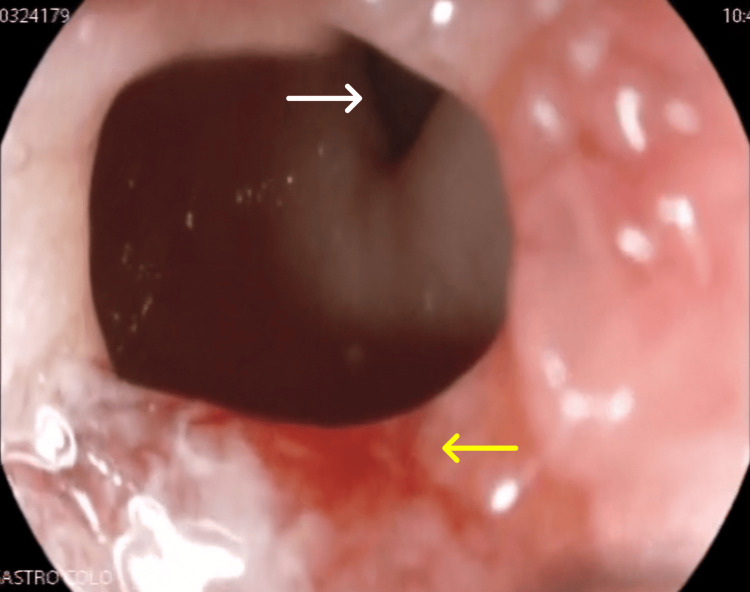
Post-balloon dilation endoscopic image demonstrating the edges of the antral web (yellow arrow), the pyloric orifice (white arrow), and the intervening chamber between the web and the pylorus

Approximately two liters of fluid were suctioned, and the antral web was successfully dilated endoscopically (Figures 3, 4), relieving the obstruction.

One month later, the patient underwent repeat gastroscopy for evaluation, which showed minimal re-narrowing of the antral web. A second balloon dilation session was performed. Follow-up gastroscopy three months later demonstrated no recurrence of the narrowing.

## Discussion

Gastric outlet obstruction due to an antral web (GAW) is a very rare entity, also known as a mucosal diaphragm of the pyloric antrum or prepyloric diaphragm, composed of mucosal layers without involvement of the muscularis propria. Although earlier cases had been reported, the condition was more clearly characterized as a distinct entity in 1977 [5].

GAW occurs predominantly in children, with the first adult case reported in 1949 [6]. In pediatric patients, symptoms may include vomiting, abdominal distention and pain, hematemesis or melena, failure to thrive, and, in some cases, the condition may be asymptomatic [7]. In adults, clinical manifestations include dysphagia or odynophagia, abdominal distention and pain, early satiety, weight loss, chest pain, and watery diarrhea, with symptoms typically exacerbated by food intake [6].

Proposed mechanisms for the development of an antral web include intrauterine vascular insult, failure of recanalization, or excessive localized endodermal proliferation [8]. In addition, a partially obstructive congenital antral web can be misdiagnosed as a peptic stricture, and superimposed ulcers may aggravate the obstruction [9]. One theory suggests that GAW may result from scar tissue formation during the healing of ulcers in the pyloric and prepyloric areas; this is supported by the prevalence of gastroesophageal reflux disease (GERD) and peptic ulcer disease (PUD) in patients diagnosed with an antral web [6].

Diagnosis can be established by upper endoscopy or an upper gastrointestinal series (UGIS). On UGIS, a characteristic double-bubble appearance may be observed, created by the antral chamber located between the web and the pylorus, with a normally appearing duodenal bulb [10]. Upper endoscopy typically reveals a smooth, diaphragm-like membrane with a fixed-size central opening and normal peristaltic movement distal to the lesion [11]. Abdominal ultrasound may also aid in the diagnosis, usually demonstrating four key findings: an echogenic diaphragm-like structure within the antrum, gastric distention, delayed gastric emptying, and a normal pylorus [11].

In their study, Salah and Baron supported the use of endoscopic therapy as the first-line treatment for selected patients with GAW [1]. Endoscopic management of symptomatic GAWs includes balloon dilatation, needle-knife incision, or repeated electrocautery cuts [4]. According to recent reports, combining balloon dilatation with needle-knife incision appears to have a low risk of perforation or major bleeding. Pre-procedural assessment with gastroscopy and endoscopic ultrasound is advised to evaluate the size of the aperture, as well as the location and thickness of the web [1].

Surgical resection remains a valuable alternative, especially in the presence of specific anatomical challenges such as an uneven or non-perpendicular mucosal layer or involvement of vascular structures or muscular layers [12]. The most frequently performed surgical techniques include exploratory laparotomy or duodenotomy with incision of the GAW at its central aperture, widening of the opening via transverse gastroplasty or pyloroplasty, or digital disruption of the web combined with pyloromyotomy [6]. In neonates and infants, web excision or incision with or without pyloroplasty is the surgery of choice [13].

Endoscopic management safely and effectively relieves symptoms of gastric outlet obstruction, and surgical treatment is now less commonly required in adults than it was previously [6]. In pediatric antral web cases, surgical treatment achieves cure in most patients and remains the standard approach, as endoscopic intervention in children is technically challenging and less well established. In the series by Yeh PJ and Chao HC, endoscopy was performed in only a small minority of cases, whereas surgery provided definitive management for nearly all patients requiring intervention [8].

## Conclusions

In conclusion, GAW is a rare cause of gastric outlet obstruction in adults. Although it is more commonly described in the pediatric population as a congenital anomaly, its occurrence in adults is uncommon and often underrecognized. The clinical presentation in adults is usually nonspecific, which increases the risk of delayed diagnosis or misdiagnosis. A high index of suspicion is required, particularly in patients with persistent obstructive symptoms and inconclusive initial evaluations. Upper endoscopy remains the gold standard for diagnosis, as it allows direct visualization of the membranous web and assessment of its location, thickness, and associated gastric lesions.

In adults, endoscopic management is considered the first-line treatment. Techniques such as endoscopic balloon dilation (EBD) and needle-knife incision have shown favorable outcomes with low morbidity. Surgical intervention is now rarely required and is generally reserved for refractory cases or when endoscopic treatment fails. Overall, although rare in adults, GAW should be considered in the differential diagnosis of gastric outlet obstruction, as timely recognition allows for effective and minimally invasive management.
